# Expression of vascular endothelial growth factor (VEGF)-C in preoperative biopsy specimens and metastatic foci of regional lymph nodes in submucosal gastric carcinoma

**DOI:** 10.1186/1477-7819-3-2

**Published:** 2005-01-07

**Authors:** Makoto Ishikawa, Joji Kitayama, Shinsuke Kazama, Hirokazu Nagawa

**Affiliations:** 1 Department of Surgery, Division of Surgical Oncology, The University of Tokyo, 7-3-1 Hongo, Bunkyo-ku, Tokyo 113-8655, Japan

## Abstract

**Background:**

Vascular endothelial growth factor (VEGF)-C is implicated in lymphangiogenesis, however the exact role of VEGF-C in promoting lymphatic spread of cancer cells remains largely unknown.

**Methods:**

The expression of VEGF-C was immunohistochemically determined in 97 endoscopic biopsy specimens from 46 patients with submucosal gastric carcinoma (SGC). Nodal metastases including micrometastasis and isolated tumor cells (ITC) were evaluated by immunohistochemical staining for cytokeratin in 1650 lymph nodes, and tumor cells in these metastatic nodes were also examined for VEGF-C expression.

**Results:**

In biopsy samples, VEGF-C was positively detected in 21 (47%) patients. Metastases were identified in 46 (2.8%) nodes from 15 (33%) patients. Metastases were detected in 39 nodes by hematoxylin-eosin (H&E) staining and in additional 7 nodes as ITC by immunohistochemical staining. The rate of lymph node metastases was significantly correlated with VEGF-C expression in biopsy samples (p < 0.05). The positive and negative predictive values of VEGF-C in biopsy specimens for nodal metastasis were 44 %(10/21) and 80% (20/25), respectively. Among the 46 metastatic nodes, tumor cells in 29 (63%) nodes positive patients expressed VEGF-C, whereas those in 17 (37%) nodes did not. VEGF-C expression was high in macronodular foci in medullary areas, whereas more than half of ITC or micrometastasis located in peripheral sinus lacked the expression of VEGF-C.

**Conclusions:**

Despite the significant correlation, immunodetcetion of VEGF-C in endoscopic biopsy specimens could not accurately predict the nodal status, and thus cannot be applied for the decision of the treatment for SGC. VEGF-C may not be essential for lymphatic transport, but rather important to develop the macronodular lesion in metastatic nodes.

## Background

The incidence of early gastric carcinoma defined as being confined to the mucosa or submucosal layer has increased. In Japan, endoscopic mucosal resection (EMR) is now generally accepted for intramucosal cancers that are associated with a minimal risk of regional lymph node (LN) metastasis [1-4]. For submucosal gastric carcinoma (SGC), however, conventional gastrectomy with complete lymph node dissection has been performed as standard treatment as the frequency of lymph node metastasis is 10–20%, which cannot be ignored clinically [5-8]. This indicates that the conventional operative procedure provides no benefit for the majority of patients with SGC, and thus criteria to safely avoid unnecessary lymphadenectomy for submucosal cancer need to be determined.

VECF-C is known to bind VEGFR-3, which is specifically expressed on lymphatic vessels and stimulates lymphangiogenesis [9,10]. Many previous reports have shown that expression of VEGF-C in cancer tissues has a positive correlation with the risk of lymphatic metastasis in breast [11,12], lung [13], colorectal [14-17], pancreatic [18], prostate [19], esophageal [14,20] and head and neck cancers [21,22]. A similar tendency has been reported for gastric cancer, although a significant correlation between VEGF-C expression and the frequency of nodal metastasis is not always found [23-27].

Recently, small metastatic lesions have been detected genetically or immunohistochemically in various cancers, even though they were diagnosed as negative by conventional examination with H&E staining. Such lesions are designated as micrometastasis or isolated tumor cells (ITC). The biological and clinical significance of such minute nodal invasion of carcinoma cells is still controversial [28-38]. In this study we performed immunohistochemical staining and extensively examined VEGF-C expression in biopsy samples and metastatic lymph nodes including micrometastasis and ITC. From these data, we attempted to determine whether the detection of VEGF-C in biopsy samples could be a clinical predictor of accurate nodal status in SGC. Then, we discussed how the VEGF-C expressed in tumor cells functions in the metastatic process to regional lymph nodes.

## Patients and methods

Forty-six patients with SGC diagnosed and treated by curative gastrectomy with standard lymph node dissection at the First Department of Surgery, Tokyo University Hospital, Tokyo, between 1994 and 2002 were included in this study. These patients were examined endoscopically prior to surgery; several pieces of tissue specimens were then sampled with routine biopsy forceps from various portions of the tumor. Formalin-fixed and paraffin-embedded sections of 97 biopsy specimens and 1650 dissected lymph nodes derived from these 46 patients were evaluated in this study. Additionally, all the resected primary tumors were histologically examined with H&E staining according to the Japanese Classification of Gastric Carcinoma [39]. Tumors were histologically classified into two types based on the predominant features: differentiated type (well and moderately differentiated adenocarcinoma) and undifferentiated type (poorly differentiated adenocarcinoma and signet ring cell carcinoma). Several discrete histological parameters, including lymphatic invasion, venous invasion and lymph node metastasis, were also evaluated.

### Immunohistochemical study of VEGF-C and Cytokeratin

The expression of VEGF-C was investigated with immunohistochemical staining using affinity purified goat polyclonal antibodies against VEGF-C (IBL, Fujioka, Japan). Sections (3-μm thick) of biopsy samples were deparaffinized in xylene, hydrated through a graded series of ethanol, and then immersed in 3% hydrogen peroxide in 100% methanol for 30 min to inhibit endogenous peroxidase activity. To activate the antigens, the sections were boiled in 10 mM citrate buffer, pH 6.0 for 30 minutes. After being rinsed in phosphate-buffered saline (PBS), the sections were incubated with normal rabbit serum for 10 min, and then incubated overnight at 4°C in humid chambers with the primary antibody to VEGF-C at 1/30 dilution. After three washes with PBS, the sections were incubated with biotinylated rabbit anti-goat immunoglobulin for 20 minute. After washing again with PBS, the slides were treated with peroxidase-conjugated streptavidin for 20 minutes, and developed by immersion in 0.01% H_2_O_2 _and 0.05% diaminobenzidine tetrahydrochloride for 3 minute. Light counterstaining with Mayer's hematoxylin was performed. The 46 lymph nodes that showed the presence of carcinoma were also evaluated for the expression of VEGF-C with the same immunostaining method.

The dissected lymph nodes were fixed in 10% formalin and embedded in paraffin. From each node, one 3-μm-thick section was prepared for H&E staining, and another three serial 5-μm sections were prepared for immunohistochemical staining with CAM 5.2 (Becton Dickinson, San Jose, CA), a mouse monoclonal antibody that reacts with human cytokeratin numbers 8 and 18 [40]. The streptavidin biotin immunoperoxidase technique was used. Deparaffinized and rehydrated sections were trypsinized with 1% calcium chloride solution at 37°C for 20 minutes. After nonspecific reactions were blocked with 10% normal rabbit serum, the sections were incubated with CAM 5.2 diluted 1/6, biotinylated rabbit anti-mouse immunoglobulin, and streptavidin peroxidase. Between each two incubation steps, sections were washed carefully in phosphate buffered saline.

### Definition of lymph node metastasis

Metastasis was defined as the presence of tumor cells, whether single or in small clusters detected by H&E or immunohistochemical staining. Metastatic lesions that were more than 2.0 mm in diameter were defined as macrometastasis, while micrometastasis was defined as a tumor deposit larger than 0.2 mm but less than 2.0 mm, and ITC was defined as a tumor deposit less than 0.2 mm in maximum diameter.

### Statistical analysis

All statistical calculations were carried out using StatView-J 5.0 statistical software (SAS Institute, USA). The relationship between clinical and pathological characteristics of patients and the expression of VEGF-C was examined by Fisher's exact test. Differences with a p value of less than 0.05 were considered to be statistically significant.

## Results

### Nodal status including micrometastases and isolated tumor cells (ITC) in SGC

**Figure 1:**
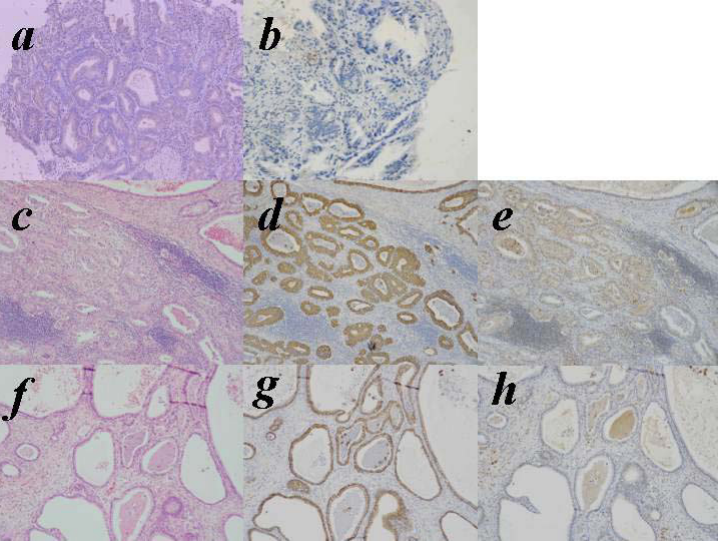
Immunohistochemical staining of VEGF-C in biopsied specimens (upper panel) and hematoxylin-eosin (H&E) staining, immunohistochemical staining of cytokeratin and VEGF-C in metastastic lymph node (middle and lower panel). ***a***, VEGF-C positive type in biopsied specimens. ***b***, VEGF-C negative type in biopsied specimens. ***c***, *f*, hematoxylin-eosin (H&E) staining in metastastic lymph node. ***d***, *g*, immunohistochemical staining of cytokeratin in metastastic lymph node. ***e***, VEGF-C positive type in metastastic lymph node. *h*, VEGF-C negative type in metastastic lymph node.

Metastases were observed in 12 patients (26.1%) and 39 lymph nodes (2.4%) by H&E examination (Fig. 1-c, f). Among these nodes, cancer cells were detected as micrometastasis (less than 2.0 mm) in 5 lymph nodes and as ITC (less than 0.2 mm) in another 5 nodes. By immunohistochemical staining with anti-cytokeratin antibody, we additionally identified such small metastatic lesions in 7 nodes (Fig. 1-d, g). All of the 7 metastatic lesions were categorized as ITC. Four nodes were derived from 4 patients who had other metastatic nodes detected by H&E staining. Cytokeratin-positive cells were also identified in other 3 nodes from 3 patients who showed no metastatic nodes by H&E staining and were diagnosed as node negative cases. Thus, the final frequency of lymph node metastasis increased to 15 of 46 patients (33%) and 46 of 1650 lymph nodes (2.8%).

Table 1 show the relationship between the exact nodal status and other clinical/pathological features. Metastases were frequently observed in tumors with deep submucosal invasion or lymphatic involvement, which were consistent with previous reports.

**Table 1:** Nodal metastasis, including nicrometastasis/ITC and clinical and/or pathological findings

	**Patients with lymph node metastasis**		
	
	**positive(15)**	**negative(31)**	**p value**
Age			
60<	6	16	
<59	9	15	0.46
Sex			
Male	11	22	
Female	4	9	0.87
Tumor size (cm)			
3.0<	11	19	
2.9>	4	12	0.42
Macroscopic type			
elevated	2	1	
depressed	13	30	0.19
Depth of invasion			
sm1	3	18	
sm2,3	12	13	0.02
Histological type			
differentiated	8	14	
undifferentiated	7	17	0.6
Lymphatic involvement			
positive	4	2	
negative	11	29	0.05
Venous involvement			
positive	2	2	
negative	13	29	0.44

### Immunohistochemical analysis of VEGF-C in biopsy and surgical specimens

The biopsy specimens were divided into two categories by the staining pattern of VEGF-C, diffuse or focal staining of carcinoma cells as described previously [26]. When distinct staining of the cytoplasm was observed in the majority of tumor cells, whether diffuse or focal, these samples were categorized as VEGF-C positive in this study (Fig. 1-a). Whereas other cases in which only a few carcinoma cells stained faintly were classified as VEGF-C negative (Fig. 1-b).

Among the 97 biopsy samples from 46 patients, carcinoma cells were contained in only one biopsy sample in 14 patients, and in 2, 3 and 4 biopsy samples in 16, 13 and 3 patients, respectively. In all of the latter cases, carcinoma cells in biopsy samples derived from different places showed exactly the same staining pattern of VEGF-C, and thus VEGF-C-positive and -negative tumors could be clearly distinguished.

In biopsy specimens, VEGF-C was positively detected in 21 (46%) cases. As shown in Table 1, the expression of VEGF-C in biopsy samples showed a significant correlation with that in surgically removed specimens (p = 0.005). However, 15 (60%) of 25 cases that were classified as VEGF-C negative in biopsy samples showed positive expression of VEGF-C in surgical specimens. In contrast, 20 of 21 cases with VEGF-C-positive biopsy samples also expressed VEGF-C in surgical specimens, indicating that positive expression of VEGF-C was mostly consistent between biopsy and surgical specimens. Thus, the immunodetection of VEGF-C in biopsy sample showed 95% of positive predictive value and 40% of negative predictive values for VEGF-C expression in primary tumor.

### Immunohistochemical detection of VEGF-C in biopsy specimens

Nodal metastasis was detected in 10 (48%) of 21 VEGF-C-positive tumors, and the rate was significantly higher than that in VEGF-C-negative tumors as evaluated by biopsy specimens (5/25, 20%) (p = 0.047). However, the positive and negative predictive values of VEGF-C in biopsy for nodal status were 44% (10/21) and 75% (20/25), respectively, and 5 (20%) of 25 VEGF-C-negative tumors were accompanied with lymph node metastasis.

### Expression of VEGF-C in tumor cells in metastatic lymph nodes

Table 3 shows the expression pattern of VEGF-C of tumor cells in 46 metastatic lymph nodes as well as in 23 biopsy samples in 15 patients. Interestingly, the expression of VEGF-C in metastatic tumor cells in lymph nodes was not necessarily correlated with that in biopsy samples. In 15 cases with nodal metastasis, VEGF-C was positively detected in 18 biopsy samples from 10 (67%) patients. On the other hand, VEGF-C was positive in metastatic tumor cells in 29 nodes derived from 7 (47%) patients (Fig. 1-e), while tumor cells metastasized in 17 (37%) lymph nodes derived from 11 (73%) patients were negative for VEGF-C (Fig. 1-h).

**Table 3:** The location of tumor cells in metastatic nodes and VEGF-C expression in 15 node positive patients.

**Case**	**VEGF-C expression**		definition of metastasis**	Detection method*	**location of tumor cells**
	**biopsy specimens#**	**metastatic nodes**			

**1**	-	-	ITC	H.E.	marginal
**2**	-	+	macrometastasis	H.E.	medullary
		+	ITC	H.E.	marginal
		+	macrometastasis	H.E.	medullary
**3**	-	-	macrometastasis	H.E.	marginal
		-	macrometastasis	H.E.	marginal
		-	ITC	I.H.C.	marginal
**4**	-	-	macrometastasis	H.E.	marginal
		-	macrometastasis	H.E.	marginal
**5**	+ +	+	macrometastasis	H.E.	marginal
		-	macrometastasis	H.E.	marginal
		-	macrometastasis	H.E.	marginal
**6**	+	+	macrometastasis	H.E.	medullary
		+	macrometastasis	H.E.	medullary
		+	macrometastasis	H.E.	medullary
		+	macrometastasis	H.E.	medullary
		+	macrometastasis	H.E.	medullary
		-	ITC	I.H.C.	marginal
**7**	+	+	micrometastasis	H.E.	medullary
		+	ITC	H.E.	marginal
		+	ITC	H.E.	marginal
**8**	+ +	+	macrometastasis	H.E.	medullary
		+	macrometastasis	H.E.	medullary
		+	macrometastasis	H.E.	medullary
		+	micrometastasis	H.E.	medullary
		+	micrometastasis	H.E.	medullary
**9**	+	-	ITC	H.E.	marginal
		-	ITC	I.H.C.	marginal
**10**	+ +	-	micrometastasis	H.E.	marginal
**11**	+ + +	+	macrometastasis	H.E.	medullary
		-	micrometastasis	H.E.	medullary
		-	ITC	I.H.C.	marginal
**12**	+ + +	+	macrometastasis	H.E.	medullary
		+	macrometastasis	H.E.	medullary
		+	macrometastasis	H.E.	medullary
		+	macrometastasis	H.E.	medullary
		+	macrometastasis	H.E.	medullary
		+	macrometastasis	H.E.	medullary
		+	macrometastasis	H.E.	medullary
		+	macrometastasis	H.E.	medullary
		+	macrometastasis	H.E.	medullary
		+	macrometastasis	H.E.	medullary
		+	macrometastasis	H.E.	medullary
**13**	-	-	ITC	I.H.C.	marginal
**14**	+ +	-	ITC	I.H.C.	marginal
**15**	+	-	ITC	I.H.C.	marginal

*:IHC means immunohistochemical staining with anti-cytochelatin mAb.

**:Micrometastasis is defined as tumor deposit larger than 0.2 mm but less than 2.0 mm, and ITC (isolated tumor cell) is defined as tumor deposit less than 0.2 mm in the maximum diameter. Larger deposits were classified as macrometastasis.

#: The number of +/- means the number of examined biopsy specimens obtained from different parts of the tumor.

Among 36 nodes from 10 patients who were determined as VEGF-C-positive in biopsy samples, tumor cells located in 10 (29%) nodes from 4 (40%) patients totally lacked the expression of VEGF-C. This finding clearly indicates that carcinoma cells that highly express VEGF-C are not always preferentially transported to the regional lymph nodes, even though the expression of this lymphangiogenic factor had a positive correlation with lymph node metastasis.

More interestingly, the expression of VEGF-C was strongly correlated with the size of metastatic foci and the location of carcinoma cells in metastatic nodes (Table 3). When carcinoma cells invaded the medullary area of lymph nodes, most of the tumor cells positively expressed VEGF-C (25/26, 96%). In contrast, the rate of VEGF-C-positive cells was markedly lower (4/20, 20%), when the carcinoma cells remained in the peripheral area of lymph nodes. Also, 22 (79%) of 28 macrometastases expressed VEGF-C, while 3 (60%) of 5 micrometastases and only 3 (25%) of 12 ITC were positive for VEGF-C. It is especially notable that all of the 7 ITC detected by immunostaining lacked expression of VEGF-C.

## Discussion

Many cancers metastasize to regional lymph nodes, and a positive nodal status often correlates with a poor prognosis of patients. However, the mechanisms of lymphatic metastasis have not been investigated in detail. Recent studies have demonstrated that the expression of VEGF-C is enhanced in various solid tumors, suggesting the possible contribution of VEGF-C to nodal metastasis, possibly through lymphangiogenesis [41,42]. Number of clinical studies has shown a positive correlation between VEGF-C expression and risk of lymph node metastasis in various cancers including gastric cancer [11,12]. However, all of the data were obtained in surgically resected specimens, and thus can not be used for preoperative information to determine the treatment.

In this study, therefore, we evaluated the expression of VEGF-C in biopsy samples in SGC. Our initial hypothesis was that VEGF-C expression can predict the accurate nodal status including micrometastasis/ITC, and thus may be useful to avoid the unnecessary gastrectomy in some SGC. Our results suggest that VEGF-C expression in biopsy specimens correlate with lymph node metastasis in SGC. However, the positive and negative predictive values were 44% and 80% respectively, and 20% of VEGF-C-negative tumors were node positive. This suggests that the immunodetection of VEGF-C in biopsy samples can not be used as clinical indicator to decide the treatment of SGC.

Present study provides some interesting findings on the possible role of VEGF-C in nodal metastasis. Biopsy samples were obtained at preoperative endoscopy, fixed with formalin immediately after biopsy, and thus appear to reflect the in situ expression level of VEGF-C more precisely than surgically resected specimens. In our results, VEGF-C expression in biopsy samples showed a significant correlation with that in surgical specimens with 57% sensitivity and 91% specificity. However, more than half (60%) of the tumors categorized as VEGF-C negative in biopsy specimens were positive in surgical specimens, although VEGF-C-positive tumors in biopsy samples showed a good consistency with those in surgical specimens. This raises the possibility that VEGF-C expression may be somewhat upregulated by surgical manipulation.

VEGF gene expression is regulated by a variety of stimuli, and hypoxia is known to be one of the most potent inducers of VEGF-A [43,44]. VEGF-C expression has also been reported to be enhanced by hypoxia in some reports [45,46], but not in others [47,48]. Although the detailed regulatory mechanisms of VEGF-C gene activation are not well understood, our results suggest a possibility that VEGF-C in surgical specimen may be induced by hypoxia during gastrectomy at least in some cases. This point should be included for the evaluation of the results in previous studies showing the positive correlation with nodal status.

Nonetheless, our data showed a significant correlation of VEGF-C expression in biopsy specimens with nodal metastasis, supporting a possible role of VEGF-C in lymphatic metastasis. As with hematogeneous metastasis, lymphatic metastasis of cancer cells is considered to be divided into several steps: invasion to lymphatic capillaries, movement into the lymphatic lumen with the lymphatic stream, attachment to the subcapsular sinus of lymph nodes, and invasion into the cortex. Lymphangiogenesis means the development and proliferation of new lymphatics from host vessels, but the ability of tumor cells to induce lymphangiogenesis and the presence of intratumoral lymphatic vessels are controversial. However, most malignant tumors are known to be associated with an increased number of lymphatic vessels in the peripheral area [42]. In fact, *in vivo *experiments using VEGF-C-transfected tumors have shown the same histological findings [49,50]. Since intratumoral interstitial fluid pressure is known to be higher than that in normal tissues, the hydrostatic pressure difference appears to transport the tumor cells from inside of the tumor to the peritumoral area. Therefore, the metastasis-promoting effect has been attributed to an increase and dilatation of peritumoral lymphatic capillaries. VEGF-C may facilitate metastasis by increasing the surface area of lymphatic vessels in contact with interstitial tumor cells in the area around the primary tumor site, and thus increase the chance of these cells entering the lymphatic system.

In regional lymph nodes, tumor cells are thought to reach the peripheral sinus from afferent lymphatics. In fact, many metastatic cells were detected around the sinus area unless they developed into a macronodular lesion. Recently, small lesions have been divided into two categories; micrometastasis and isolated tumor cells (ITC), which are distinguished based on their size [51]. Micrometastasis is defined as a tumor deposit larger than 0.2 mm but less than 2.0 mm, while ITC is defined as a tumor deposit less than 0.2 mm in maximum diameter. Although the biological features of these categories have not been fully clarified, they are now pathologically defined as pN1m1 and pN0, respectively.

In our study, the metastatic lesions did not always express VEGF-C, and such small metastatic foci often lacked the expression of VEGF-C. Especially, ITC identified only with immunohistochemical staining are totally negative for VEGF-C. In addition, in 4 tumors with lymphatic invasion, none of the tumor cells located in the lymphatic vessels in the primary tumor expressed VEGF-C (data not presented). These unexpected results suggest that expression of VEGF-C in tumor cells is not relevant to the transportation to regional nodes once they enter lymphatic vessels.

In contrast, most of the macrometastases or cancer cells invading the medullary area of metastatic nodes highly expressed VEGF-C. This phenomenon was quite interesting, though not fully explained by today's knowledge. This may suggest that proliferation and invasion in the internal area of metastatic nodes may partially require VEGF-C expression in tumor cells. Thus far, there is no definite report on the effects of VEGF-C on tumor cells. The VEGF-receptor 3 (VEGFR-3), a specific ligand of VEGF-C, was expressed only on certain tumor cells [52-54] and not on others. We tried to examine the expression of VEGF-C receptor in these gastric cancers using a polyclonal antibody to VEGFR-3, but could not detect positive staining in any case (data not shown). Thus, it seems to be unlikely that VEGF-C directly affects the behavior of gastric cancer cells. However, it remains a possibility that VEGF-C secreted from tumor cells may act on intranodal lymphatic endothelial cells or other interstitial cells and create favorable conditions for tumor cell growth or invasion in lymph nodes.

In summary, our retrospective study demonstrated that VEGF-C expression in tumor cells in biopsy specimens was significantly correlated with lymphatic metastasis in SGC, although the accuracy was not high enough to be used for clinical indicator. Metastatic tumor cells in micrometastasis or ITC located in marginal sinus often lacked the expression of VEGF-C, whereas macrometastasis located in the medullary area in metastatic nodes highly express VEGF-C. This suggests a possibility that expression of VEGF-C is not essential for lymphatic transport from primary tumor, but rather important to develop the macronodular lesion in metastatic lymph nodes.

## Competing Interests

The author(s) declare that they have no competing interests.

## Authors' contributions

**MI**. Conceived of the study and wrote the original version of the manuscript.

**JK**. Carried out the literature search and helped in drafting the manuscript.

**SK**. Collected clinical and pathologic data and participated in manuscript preparation

**HN **Helped to shape the idea for the study coordinated the study and edited the manuscript.

All authors have read and approved the final manuscript.

**Table 2:** Relationship between the expression of VEGF-C in surgical and biopsy specimens

	**Surgical specimens**			**Nodal metastasis**		
				
	positive (35)	negative (11)	p value	positive (15)	negative (31)	p value
**Biopsy specimens**						
positive (21)	20	1		10	11	
negative (25)	15	10	0.005	5	20	0.047

**Table 4:** The expression of VEGF-C in surgical specimens and nodal metastasis

	**Positive (35)**	**Negative (11)**	**p value**
Lymph node metastasis			
positive	14	1	
negative	21	10	0.05
